# The Effect of sp^2^ Content in Carbon on Its Catalytic Activity for Acetylene Hydrochlorination

**DOI:** 10.3390/nano12152619

**Published:** 2022-07-29

**Authors:** Fangjie Lu, Chengcheng Wei, Xue Yin, Lihua Kang, Mingyuan Zhu, Bin Dai

**Affiliations:** 1 College of Chemistry and Chemical Engineering, Yantai University, Yantai 264004, China; lufangjie2022@126.com (F.L.); yxshzu@126.com (X.Y.); zhuminyuan@shzu.edu.cn (M.Z.); 2 School of Chemistry and Chemical Engineering, Shihezi University, Shihezi 832000, China; db_tea@shzu.edu.cn; 3 Shandong National Standards Technical Review and Assessment Center, Jinan 250002, China; weichengcheng2014@163.com

**Keywords:** acetylene hydrochlorination, specific activity, carbon materials, sp^2^ ratio

## Abstract

We report the influence of sp^2^ content in carbon catalyst on the catalytic activity for acetylene hydrochlorination. Nanodiamonds (NDs) were used as the precursor and calcinated under different temperatures. The resulting ND500, ND700, ND900, and ND1100 catalysts were characterized, and the sp^2^ content increased with increasing calcination temperature. The specific activities of the catalysts first increased and then decreased with increasing sp^2^ content. The highest catalytic activity could be obtained in the ND-900 catalyst with a sp^2^ value of 43.9%. The density functional theory results showed that the adsorption sites for acetylene and hydrogen chloride were located at the interface between sp^2^ and sp^3^ configuration.

## 1. Introduction

Acetylene hydrochlorination is a very important method to produce vinyl chloride monomer (VCM) in China. Mercury chloride is used as an active component in this reaction, but the mercury component in the mercury-based catalyst is easily lost, which pollutes the environment and can harm human health [1]. The development of green, environmentally friendly, non-polluting, and high-efficiency mercury-free catalysts has drawn the attention of countries around the world.

Noble metal catalysts such as Au [1,2] and Ru [3] have been extensively investigated and exhibit excellent catalytic performance for acetylene hydrochlorination. However, large-scale commercial applications of these noble metal catalysts for acetylene hydrochlorination are still incomplete. In recent years, nitrogen-doped carbon materials have gained much attention as an effective catalyst for acetylene hydrochlorination. Generally, non-metal catalysts require more heat than metal catalysts, and their catalytic activity is comparable to that of supported metal catalysts at higher reaction temperatures (220 °C). In our previous work, we reported that active carbon (AC)-supported g-C_3_N_4_ catalyst exhibited considerable catalytic activity for acetylene hydrochlorination as a non-metallic catalyst, and nitrogen atoms served as the adsorbed site for hydrogen chloride, and acetylene was adsorbed at the carbon atom [4]. Since then, many researchers have tried to improve the catalytic activity of nitrogen-doped materials by doping with other heteroatom such as B [5], S [6], and P [7]. In general, the doping of other heteroatoms can enhance the catalytic activity due to two reasons: (i) heteroatom doping can effectively enhance the ratio of pyridine or pyrrole type nitrogen in these materials, which play an important role in the catalysis of acetylene hydrochlorination. (ii) Nitrogen atoms are lost in the heat treatment process of the catalyst and form a defective carbon ring in the nitrogen-doped materials. Therefore, many groups have synthesized pyridinic or pyrrolic N-rich carbon material to obtain an excellent catalyst for acetylene hydrochlorination.

Lin et al. presented a cutting-edge strategy that allowed systematic tuning of the electrical conductivity of polyaniline-derived N-doped carbons at a defined nitrogen speciation and content [8]. Other researchers tried to control the carbon defects in the nitrogen-doped carbon catalyst. Qiao et al. synthesized g-C_3_N_4_ framework with a porous structure and rich nitrogen defects; the nitrogen defects in the g-C_3_N_4_ framework greatly improved the adsorption of HCl and acetylene, and the catalyst showed a highly efficient activity, with acetylene conversion reaching 94.5% [9]. However, the catalyst with high catalytic activity needed both high nitrogen content and carbon defects, but these two factors conflict with the synthesis of carbon nitrogen materials [10] because nitrogen atoms can be oxidized to NOx gas and run off in the high-temperature calcination process; calcination is necessary for the formation of carbon defects in these materials. Therefore, a pure carbon catalyst without nitrogen doping may be another strategy for the development of non-metal catalyst for acetylene hydrochlorination.

In our previous work, we summarized that the defects in graphene could improve catalytic performance based on density functional theory (DFT)-calculated results [11]. Li et al. reported that a ND-graphene material composed of a ND core and a defective graphitic shell exhibited an acetylene conversion of 50% at 220 °C. The defects on the carbon could serve as efficient catalytic active sites for acetylene hydrochlorination [12]. The surface defects can be improved by removing surface oxygen groups directly or first oxidizing them by a nitrate solid-phase oxidative method followed by thermal treatment; catalytic performance is linearly correlated with the extent of surface defects in carbon [13]. It is well-known that the defect in carbon materials is related to their physical properties, such as edges, rings, sp^2^/sp^3^ ratio, and dopant. The defects in carbon significantly affect the catalytic performance of carbon in other catalysis research areas [14,15]. However, the relationship between the carbon matrix and the catalytic activity of acetylene hydrochlorination remains confusing and controversial, which is important for designing non-metal catalysts with enhanced catalytic activity for acetylene hydrochlorination.

Different carbon allotropes, such as graphene [16], carbon nanotube [17], and carbon nanoflowers [18], have been reported to be active for the catalysis of acetylene hydrochlorination. The carbon atom after sp^2^ hybridization exists in a two-dimensional planar structure, and the active site is produced by changing the charge density at the carbon atom or C-C bond [19]. Therefore, we suspect that the way carbon atoms hybridize can change the electronic cloud arrangement of carbon materials, which may affect their catalytic performance for acetylene hydrochlorination. In this work, we try to investigate the effect of sp^2^ content in carbon materials on the catalytic performance for acetylene hydrochlorination; this investigation provides some essential insights for targeted optimization, which are fundamentally important and technically promising to accelerate the development of non-metal catalysts for acetylene hydrochlorination.

## 2. Experimental Section

### 2.1. Material

Nanodiamonds (NDs) were purchased from Beijing Grish Hitech Co., Ltd, Beijing, China. C_2_H_2_ (acetylene gas, 99%) and HCl (hydrogen chloride gas, 99%) were also used.

### 2.2. Catalyst Preparation

First, 3 g of ND was pretreated with 30 mL of 1 M HCl solution at 80 °C for 6 h to remove impurities; then, the mixture was filtered with deionized water to neutrality. Treated ND were obtained after drying at 80 °C for 12 h. Finally, the ND were heated from 25 to 500 °C, 700 °C, 900 °C, and 1100 °C at a heating rate of 5 °C/min in an Ar atmosphere for 4 h, and the catalyst was named ND500, ND700, ND900, and ND1100, respectively.

### 2.3. Catalyst Characterization

Transmission electron microscopy (TEM) was performed using a JEM 2010 electron microscope at an accelerating voltage of 200 kV to examine the sample morphologies. The Horiba Jobin Yvon LabRAM HR800 was used to collect Raman spectra using the 633 nm laser excitation. X-ray photoelectron spectroscopy (XPS) was scanned by Thermo ESCALAB 250XI experiment with Al, Ka X-ray source (225 W). The surface area data were calculated by the Brunauer–Emmett–Teller (BET) Micromeritics ASAP 2020 with a nitrogen adsorption isotherm at 77 K. TPD (temperature programmed desorption) analysis was performed using a Chem BET Pulsar TPD/TPR (automated chemical adsorption analyzer) at a heating rate of 10 °C/min to 900 °C in an atmosphere of helium flow rate of 100 mL/min. Thermogravimetric analysis (TGA) of the catalysts was performed using a TGDSC synchronous thermal analyzer (NETZSCH STA 449 F3 Jupiter©, Selb, Germany) heated to 1000 °C in an air atmosphere.

### 2.4. Catalytic Performance Evaluation

The fixed-bed micro-reactor with 10 mm inner diameter was used to evaluate the catalyst performance. The volume and mass of the filling catalyst were 1 mL and 0.9 g, respectively. Nitrogen was used to remove air from the micro-reactor. After nitrogen purging for 30 min, the temperature was increased to 220 °C via a temperature controller (model CKW-1100). Acetylene gas (0.83 mL min^−1^) and hydrogen chloride (0.95 mL min^−1^) were then fed through the reactor at a gas hourly space velocity (GHSV, C_2_H_2_) of 50 h^−1^. The GC2014C with an FID detector was then used to analyze the reaction products. The conversion of acetylene (XA) and selectivity of the catalyst to VCM (SVC) were calculated as follows:

XA = (ΦA0 − ΦA)/ΦA0 × 100%(1)

SVC = ΦVC/(1 − ΦA) × 100%(2)

In these equations, ΦA0 is the volume fraction of acetylene in the raw gas, ΦA is the volume fraction of remaining acetylene in the product gas, and ΦVC is the volume fraction of vinyl chloride in the product gas.

### 2.5. Computational Details

The adsorption energy of hydrogen chloride and acetylene was calculated by DFT calculation. All simulations were performed using the Guassian09 software package using the mixed-density functional method M062X [20,21]. Nonlocal correlation functional with the basis set of 6–31 G (d, p) was used for hydrogen (H), carbon (C), nitrogen (N), and chlorine (Cl) atoms.

## 3. Results and Discussion

**Figure 1:**
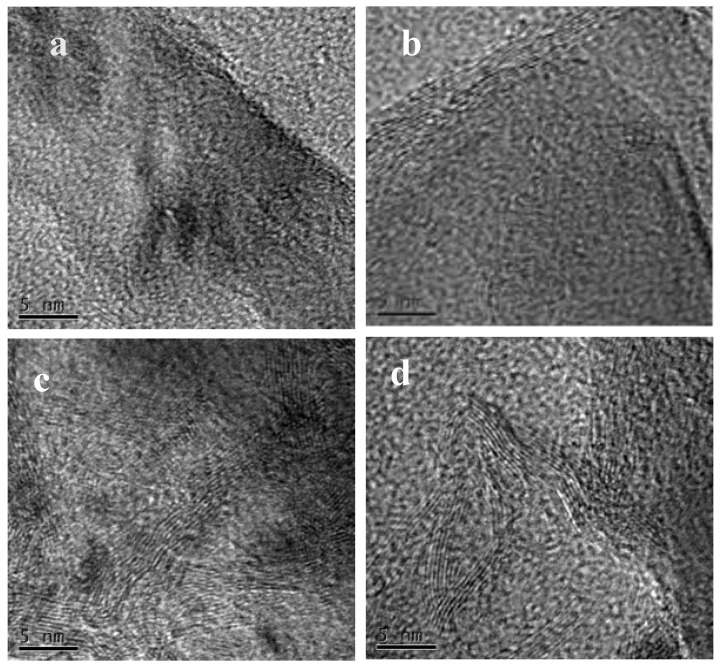
The TEM images of ND catalysts treated at different temperatures for ND500 (**a**), ND700 (**b**), ND900 (**c**), and ND1100 (**d**).

Figure 1 shows the TEM images of ND500, ND700, ND900, and ND1100 samples. After purification in strong oxidizing acid, the surface of NDs is covered with abundant oxygen groups as well as amorphous carbons. When NDs are thermally treated, the decomposition of functional group at their surface, which requires energy. The structure of nanodiamonds changes from the surface outer layer small particles to form the sp^2^ hybrid form of carbon atoms at lower pyrolysis temperature; as the pyrolysis temperature increases, the deep nanoparticles (>10 nm) require more heat to undergo a phase transition, and the sp^2^ graphite-like shells increase [22]. There is mixed sp^2^/sp^3^ bonding at the interface in the diamond core and outer layer and a sp^3^ carbon core covered with sp^2^ graphite-like shells, which belongs to the sp^2^/sp^3^ hybrid materials [23,24]. Figure 1 shows that when the temperature is lower than 900 °C degrees, the number of sp^2^ graphite-like shells is less. When the temperature is higher than 900 °C, the number of graphite shell layers can reach seven layers, the number of carbon layers increases, and multiple layers of curved sp^2^ shells can be clearly observed. Thus, the number of graphite layers of the hybrid NDs gradually increased with increasing treatment temperatures.

**Figure 2:**
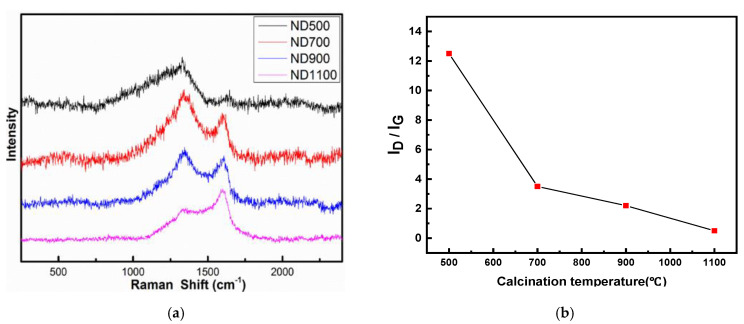
(**a**) Raman spectra of ND500, ND700, ND900, and ND1100 catalysts. (**b**) The I_D_/I_G_ data in the ND catalysts with different calcination temperatures.

Raman spectroscopy is a powerful tool for characterizing the structural transformation of catalysts. There are two distinct bands at 1336 and 1647 cm^−1^ [24], which conform to the D and G bands, respectively (Figure 2a). The relative area value ID of the D peak indicates the degree of lattice defect and disorder of the carbon material and the degree of sp^3^ hybridization. The relative area value IG of the G peak indicates the degree of graphitization of the carbon material and the degree of sp^2^ hybridization. The peak area of the G band increases significantly as the calcination temperature increases. When the calcination temperature reaches 700 °C, the graphite G-band derived from the diamond precursor appears along with a disordered D-band, meaning that the sp^3^-hybridized carbon begins to transform into a sp^2^-hybrid. Figure 2b lists the I_D_/I_G_ based on the Raman data. The G peak content increases and I_D_/I_G_ value decreases when the onset temperature (i.e., phase transition) of graphitization is usually in the range of 700–900 °C. There are many oxygen-containing functional groups on the surface of ND, and the calcination process could remove these surface contaminants and partially decompose the outer sphere of ND to form a graphitic fullerene shell, thus transforming the bulk nanocrystal into a uniform and self-assembled sp^2^/sp^3^ hybrid structure [25]. The phase change produces a sp^2^ hybridized carbon shell outside the ND and then continuously graphitizes inside the particle [26]. The ND surface and the newly created structural defects resulting from the separation of surface functional groups increase the reactive atoms of the surface carbon, thus promoting the phase during the transformation. For temperatures above 1100 °C, the highly disordered carbon shell becomes increasingly graphitized, and the defect density becomes lower and lower. Table 1 shows the I_D_/I_G_ data of each sample based on Raman analysis, I_D_/I_G_ data of nanocarbon catalysts ND500, ND700, ND900, and ND1100 are 12.5, 3.5, 2.2, and 0.5.

**Figure 3:**
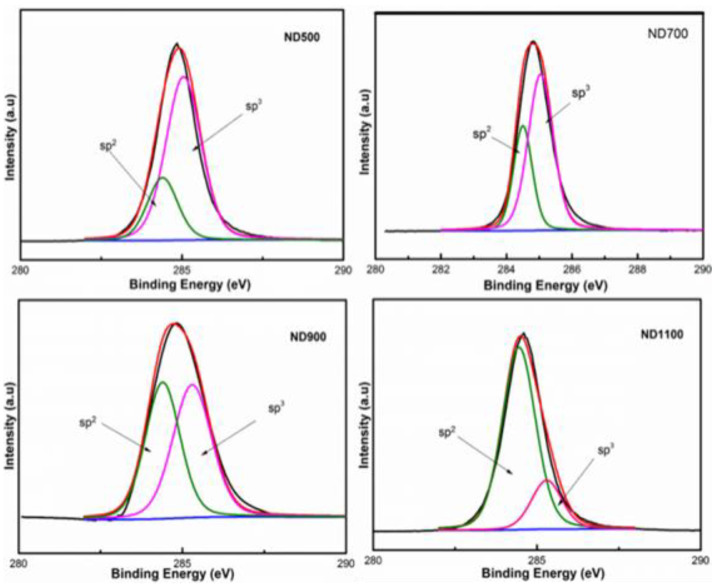
XPS deconvolution of C1s for ND500, ND700, ND900, and ND1100 catalysts.

XPS further investigated the content of sp^2^ hybrids in the catalysts. Figure 3 shows that the carbon on the catalyst mainly exists in the hybrid mode of sp^2^ and sp^3^, and the binding energy peaks correspond to 284.7 and 285.5 eV [16]. The content of sp^2^ increases with increasing heat, and the degree of graphitization became higher in the catalyst. The sp^2^/sp^3^ data of nanocarbon catalysts ND500, ND700, ND900, and ND1100 are 0.25, 0.43, 0.78, and 2.34 (Table 2). Compared with the ND900 catalyst, the sp^2^/sp^3^ data of ND1100 has a larger increase, indicating that the degree of graphitization of ND is significantly increased under the heat treatment above 1100 °C, and it has an obvious graphite-like structure. The results from XPS indicated that the sp^2^ content in the ND catalysts increased with increasing carbonization temperature. The content of sp^3^ decreases with increasing heat, and the degree of graphitization became higher in the catalyst. The results from Raman and XPS indicated that the sp^2^ content in the ND catalysts increased with increasing carbonization temperature.

It is well-known that the catalytic activity of the catalyst is related to its surface areas, so the physical structural parameters of the ND catalysts are investigated. As listed in Table 3, the specific surface of the nanocarbon catalyst gradually increases before the reaction with the carbonization temperature increment. The ND1100 expands to 340.5 m^2^/g, and the pore size and pore volume increase slightly. The increase in the calcination temperature will effectively remove functional groups with the formation of CO and CO_2_ gases [24]. The formation of CO or CO_2_ gases may produce micropores or mesopores pores in the calcination process, and this may explain the increased surface areas with calcination temperatures.

**Figure 4:**
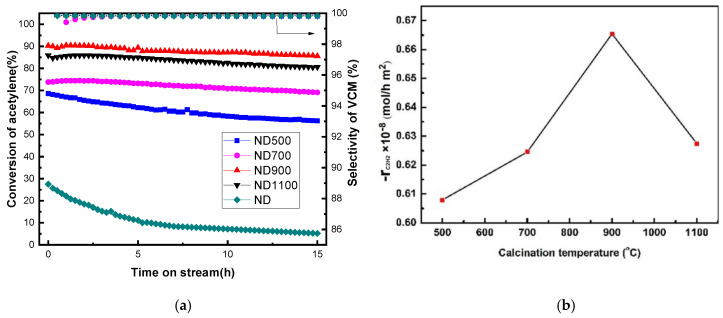
(**a**) Acetylene conversion and selectivity on the ND, ND500, ND700, ND900, and ND1100 catalysts. (**b**) The relationship between the calcination temperature and reaction rate per surface area.

The activity of the catalyst was evaluated by a fixed reactor under the following conditions (reaction temperature = 220 °C, GHSV (C_2_H_2_) = 50 h^−1^, V_HCl_/V_C2H2_ = 1.15). Combined with literature reports, ND can obtain nano-carbon catalysts with different structures after different calcination temperatures. When the calcination temperature is lower than 700 °C, sp^3^-hybridized ultra-dispersed nanodiamonds are formed. When the calcination temperature is 700–800 °C, sp^2^/sp^3^ -hybridized bucky nanodiamonds start to form. When the calcination temperature is 900–1100 °C, curved sp^2^-hybridized onion-like carbons appear [24]. Considering the temperature error range of the tube furnace instrument itself, the calcination temperature was selected as 500 °C, 700 °C, 900 °C, and 1100 °C to achieve the preparation of NDs catalysts with different structures. Figure 4a shows that the initial activities of the ND, ND500, ND700, ND900, and ND1100 can reach 28.2, 68.5, 75.5, 90.6, and 86.5%, respectively. The acetylene selectivity of a series of catalysts is all above 99.5%, and no byproduct materials are generated. The ND catalyst treated with acid alone was almost inactive after 15 h of reaction, indicating that the poor activity of the uncalcined ND catalyst was due to the sp^3^ hybridization of its own carbon. The ND900 catalyst exhibits the best catalytic activity relative to the other three catalysts. We found that when the calcination temperature was increased, the surface area of the catalyst increased, and when the adsorption amount of the reaction gas increases, and the catalytic activity increases. Therefore, to deduct the influence of the surface area on the activity, we propose the concept of specific activity; that is, the molar amount of acetylene converted per unit area and unit time determines the catalytic activity. Based on the intimal conversion of acetylene and the surface areas of these four catalysts, we calculated the specific activity of NDs according to the following Equation (3) [27]:

(3) $$r=\frac{q\cdot X_{A}}{22400\cdot S\cdot W}$$

Here, *q* is the flow of acetylene gas, *X_A_* is the conversion of acetylene on NDs, *S* is the surface area of NDs, and *W* is the mass of the filling catalysts in the fixed bed reactor. As shown in Figure 4a, the highest conversions *X_A_* of nanocarbon catalysts ND500, ND700, ND900, and ND1100 are 68.5, 75.5, 90.6, and 86.5%. Figure 4b shows that the specific activity gradually increased with the increase of sp^2^ content in the catalyst. When the calcination temperature was 900 °C, the ND900 catalyst displayed the best specific activity, and the specific activity of ND1100 is lower than that of ND900. This result means that the content of sp^2^ greatly affects the catalytic activity of the ND catalysts. The catalytic activity of carbon increases with increasing sp^2^ content when the sp^2^ content is lower than the value of 43.9% (i.e., the sp^2^/sp^3^ is 0.78) in ND900, indicating that the presence of graphene carbon with sp^2^ hybridization can enhance the catalytic activity of carbon catalyst; this is consistent with the recent literature [28]. The catalytic activity of the carbon catalyst is lower when the sp^2^ content is 70.1% (i.e., the sp^2^/sp^3^ is 2.34). This indicates that there is an optimal sp^2^ content in the structure of carbon, and we infer that the catalytic performance is increasingly dependent on the presence of a mixed sp^2^/sp^3^ configuration rather than the separate sp^2^ or sp^3^ hybrid.

**Figure 5:**
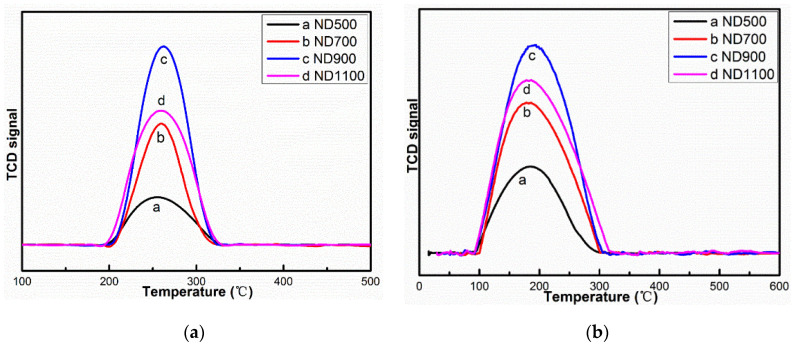
TPD evolution profiles of (**a**) acetylene and (**b**) hydrogen chloride.

The TPD of acetylene and hydrogen chloride reactants is shown in Figure 5. The desorption peaks of acetylene are located at about 260 °C, and these peaks of hydrogen chloride were located near 170 °C, which is similar to the literature [4]. The desorption temperature of acetylene was higher than that of hydrogen chloride, which means that the adsorption of acetylene was stronger than hydrogen chloride. Acetylene was first adsorbed on the active sites of NDs catalysts, and then, hydrogen chloride adsorbed and reacted to produce VCM in the acetylene hydrochlorination process. The desorption peaks of both acetylene and hydrogen chloride followed the sequence of ND900 > ND1100 > ND700 > ND500. We believe that the adsorption is affected by the combination of specific surface area and sp^2^/sp^3^ content. According to Table 3 and Figure 4a and Figure 5, as the surface area increases, the adsorption increases and the catalytic activity increases. We subtracted the effect of surface area to obtain specific activity data (Figure 4b), and it was found as shown in Figure 5 that the adsorption of NDs catalyst for acetylene and hydrogen chloride is consistent with the acetylene conversion and specific activity in Figure 4. We conclude that the specific activity of NDs may be related to the adsorption of acetylene and hydrogen chloride. However, it also shows that the adsorption is affected by the sp^2^/sp^3^ content. When it is higher than sp^2^/sp^3^ = 0.78, the amount of adsorption decreases and the catalytic activity decreases. Therefore, it shows that the ND900 catalyst with the best sp^2^/sp^3^ = 0.78 has the strongest adsorption for the reactants.

**Figure 6:**
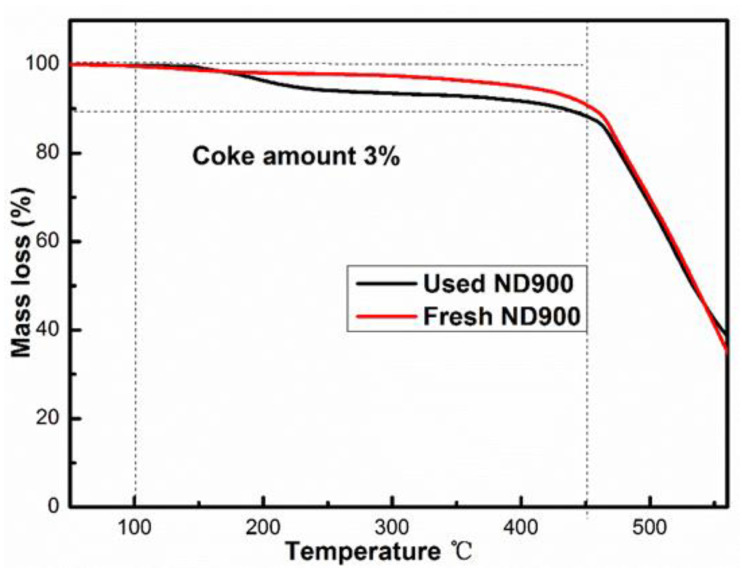
Thermogravimetric analysis (TGA) curves recorded in an air atmosphere of fresh and used ND900 catalyst.

To explore the reason for the deactivation of the prepared ND900 catalyst, we conducted thermogravimetric analysis of the ND900 catalyst before and after the reaction, and the results are shown in Figure 6. As shown in Figure 6, in the range of 100–450 °C, the weight loss is the amorphous carbon burned and decomposed on the surface. The carbon deposition of the catalyst before and after the reaction is 3%, which indicates that the surface of the catalyst ND900 has carbon deposition. It also shows that the reason for the rapid deactivation of the catalyst with the acetylene hydrochlorination reaction is carbon deposition. It shows that with the progress of the reaction, the acetylene and vinyl chloride oligomers have carbon deposits on the catalyst surface to cover the active sites of the carbon material itself, thereby reducing the catalytic activity of the ND900 carbon material.

**Figure 7:**
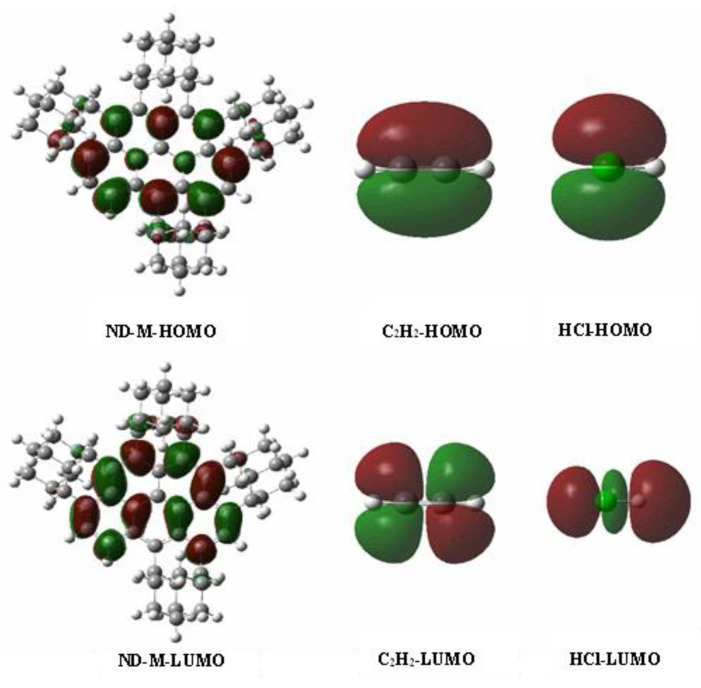
Frontier molecular orbitals of HOMO and LUMO for ND-M, C_2_H_2_, and HCl.

In order to further investigate the sp^2^ and sp^3^ hybrid effect on acetylene hydrochlorination catalysis, we built a model with five aromatic rings and four adamantanes (Figure 7). The sp^2^ ratio was about 43.9%, which is similar to the value of the ND-900, which exhibited the best catalytic activity for acetylene hydrochlorination. Table 2 lists the energy details and the graph of frontier molecular orbital (FMO) for the ND-M catalyst. The energy gaps indicate the ability of electrons to transfer between C_2_H_2_/HCl molecules and the catalyst. In Table 4, the energy gaps of the HOMO–LUMO (C_2_H_2_/HCl → ND-M) are larger than those of the HOMO–LUMO (ND-M → C_2_H_2_/HCl). This difference suggests that the catalyst is an electron donor, the C_2_H_2_ and HCl molecules are the electron accepter, and the electron shift occurs from the HOMO of the catalyst to the LUMO of the C_2_H_2_ and HCl molecules.

In the single adsorption structure of C_2_H_2_ and HCl molecules, both C_2_H_2_ and HCl are negatively charged with charges at −0.0103 and −0.00705 e, respectively. Due to the molecular orbitals obtained by overlapping, the same phase can accumulate electrons in the region between atoms, thus forming stable bonds between atoms and forming molecular bonding orbitals. The electron transfer of HOMO (catalyst) to LUMO (C_2_H_2_) indicates that the maximum overlap between the LUMO of C_2_H_2_ and the HOMO of the catalyst in the same phase is required, and the optimal adsorption site is located at the lower left of the catalyst. Furthermore, the corresponding orbital energies of the ND-M-C_2_H_2_ complexes are calculated to investigate the interactions between ND-M-C_2_H_2_ complexes and HCl molecule. The HOMO-LUMO energy gaps of (ND-3-C_2_H_2_→HCl) are smaller than (HCl → ND-3-C_2_H_2_), indicating that HCl accepts electrons and the ND-3-C_2_H_2_ complex donor electrons. The electrons transfer from the HOMO of ND-3-C_2_H_2_ complexes to the LUMO of the HCl molecule.

## 4. Conclusions

In summary, the effect of sp^2^ hybrid content in the carbon catalyst on the catalytic activity for acetylene hydrochlorination was investigated in this work. The catalytic activity first increased and then decreased with increasing sp^2^ content in the carbon. The optimal sp^2^ content in ND900 was about 43.9%. The ND900 catalyst also displayed the best adsorption for both acetylene and hydrogen chloride. FMO on the catalyst and reactants showed that the electron transfer occurred on the conjunction of the sp^2^ and sp^3^ structure, and the interface between the two-dimensional planar structure and the three-dimensional spatial structures in the carbon may provide active sites for the catalysis of acetylene hydrochlorination. The results may provide a new strategy for the design of carbon catalysts for acetylene hydrochlorination.

## Figures and Tables

**Table 1:** The I_D_/I_G_ data of the samples based on Raman.

Catalyst	Area%		I_D_/I_G_
	D	G	
ND500	92.6	7.4	12.5
ND700	77.8	22.2	3.5
ND900	69.0	31.0	2.2
ND1100	34.2	65.8	0.5

**Table 2:** The sp^2^/sp^3^ data of the samples based on XPS.

Catalyst	Area%		sp^2^/sp^3^
	sp^2^	sp^3^	
ND500	20.2	79.8	0.25
ND700	30.1	69.9	0.43
ND900	43.9	56.1	0.78
ND1100	70.1	29.9	2.34

**Table 3:** BET analysis of ND500, ND700, ND900, and ND1100 catalysts.

Catalyst	S_BET_ (m^2^ g^−1^)	V (cm^2^ g^−1^)	D (nm)
ND500	258.3	0.22	3.7
ND700	303.5	0.26	3.6
ND900	336.3	0.29	3.6
ND1100	350.5	0.30	3.7

**Table 4:** The energy detail values of the ND-M catalyst.

	HOMO → LUMO					
	HOMO	LUMO	C_2_H_2_/HCl	Cat→	HCl→	(Cat-C_2_H_2_)
			→Cat	C_2_H_2_/HCl	(Cat-C_2_H_2_)	→HCl
ND-M	0.140	−0.026	-	-	-	-
C_2_H_2_	−0.286	0.050	−0.259	−0.190	-	-
HCl	−0.333	−0.030	−0.307	−0.136	-	-
ND-M-C_2_H_2_	0.142	−0.028	-	-	−0.305	−0.138

## Data Availability

Not applicable.
